# Dr. George Leroy Rice

**Published:** 1912-01

**Authors:** 


Dr. George Leroy Rice, retired, died at Salamanca, November 27, 1911.

